# Assessing the Effect of Diesel Fuel on the Seed Viability and Germination of *Medicago sativa* Using the Event-Time Model

**DOI:** 10.3390/plants9091062

**Published:** 2020-08-19

**Authors:** Michael O. Eze, Grant C. Hose, Simon C. George

**Affiliations:** 1 Department of Genomic and Applied Microbiology and Goettingen Genomic Laboratory, Georg-August University of Goettingen, 37077 Goettingen, Germany; 2 Department of Earth and Environmental Sciences and MQ Marine Research Centre, Macquarie University, Sydney, NSW 2109, Australia; simon.george@mq.edu.au; 3 Department of Biological Sciences, Macquarie University, Sydney, NSW 2109, Australia; grant.hose@mq.edu.au

**Keywords:** phytoremediation, diesel fuel, *Medicago sativa*, germination, seed viability, event-time model

## Abstract

The remediation of contaminated sites using plant-based techniques has gained increasing attention in recent decades. However, information on the effects of contaminant imbibition on seed viability and germination rates are often lacking in the literature. To this end, our research investigated, by means of an event-time model, the effect of diesel fuel imbibition on the seed viability and germination rate of *Medicago sativa*, a plant species with great potential for remediation of organic contaminants. The event-time model provided an accurate and biologically relevant method for analysing germination data. Our results reveal that the direct imbibition of diesel fuel by *M. sativa* seeds for ≤48 h, or their exposure to soil diesel fuel concentrations of 0–10 g/kg diesel fuel, affects their germination rates, as shown by increasing *t_50_* values from 90.6 (±2.78) to 114.2 (±2.67) hours, without significantly affecting seed viability. On the other hand, diesel fuel imbibition of longer duration, or the exposure of *M. sativa* seeds to ≥20 g/kg diesel fuel-contaminated soils, leads to no further effect on time to seed emergence. Instead, these conditions compromise seed viability, resulting in a decrease in the proportion of germinated seeds from 0.91 (±0.03) in 10 g/kg diesel fuel contaminated soil to 0.84 (±0.04) and 0.70 (±0.05) in 20 and 30 g/kg diesel fuel-contaminated soils, respectively. The fact that low concentrations of diesel fuel and 0–48 h of direct imbibition delayed seed emergence without adversely affecting the percentage of viable seeds suggests that this inhibitory effect on germination at low diesel fuel exposure could be attributed more to physical constraints rather than biological damage on the seeds. The models used in this study provide an accurate and biologically relevant method for the analyses of germination data. This is vital since expensive germination experiments, be it in the field of toxicology or agriculture, deserve to be accurately analysed.

## 1. Introduction

There is a growing body of evidence that plant roots, in conjunction with their associated microbial communities, offer an effective treatment strategy for in situ remediation of contaminated soils [1,2]. Under a variety of environmental conditions, vegetation has been shown to enhance microbial degradation rates of organic chemical residues in soils [3]. Plant-based remediation (phytoremediation) is not a new concept. Constructed wetlands, reed beds and floating-plant systems have been widely used for the treatment of some types of wastewater. Current research now focuses on expanding phytoremediation to address contaminated soils and atmospheric pollutants [4]. These techniques provide environmentally friendly and cost-efficient advantages over excavation and off-site treatments of contaminated soils.

The focus of recent advances in bioremediation techniques has been to effectively restore polluted environments in an eco-friendly manner, and at a low cost [5]. To achieve this goal, various laboratory and greenhouse-based studies have been performed to assess the suitability of plant species for phytoremediation [6,7,8]. In many of these experiments, plants are first grown in uncontaminated soils for a week or two and then transplanted to contaminated soils. Hence, there is a dearth of literature on the effects of contaminant imbibition on seed viability and germination rates—something necessary to complement microcosm studies on phytoremediation of organic contaminants. The aim of the present study is to bridge this gap by examining the effect of exposure to diesel fuel on the seed viability and germination rate of *Medicago sativa*, a plant with great reclamation potential for soils contaminated with petroleum hydrocarbons [9,10,11,12,13,14].

Diesel fuel is hydrophobic [15], and in low permeability matrices such as soil will tend to not migrate into deeper sediments. Therefore, the majority of the hydrocarbon contaminants from diesel spills will be held within the surface and subsurface layers of soils, and within the rhizospheric zones of plants. As a result, seeds planted in such soils either for agricultural purposes or for the purpose of remediation and land reclamation will come into direct contact with diesel fuel and become coated by it. Thus, it is imperative that the effect of such hydrocarbon contaminants on seed viability and germination be properly understood.

Germination experiments can be divided into two groups: those in which new batches of seeds are used for each test, and those in which the same batch of seeds is followed over time. In the latter case, the same seeds are repeatedly observed over a pre-specified duration of the experiment until the event of interest occurs; the resulting data are often referred to as time-to-event data [16]. Time-to-event data have two inherent features. Firstly, the event of interest need not occur at all during the experiment. This phenomenon, known as right-censoring [17], is applicable to germination experiments since some seeds may not germinate for the entire duration of the experiment. Consequently, a plausible statistical model must allow for the event of interest (in this case, seed germination) occurring after termination of the experiment, or not occurring at all. Secondly, the event of interest may not be observed exactly at the time point when the event took place [17]. For instance, seeds in pots or petri dishes may only be inspected once a day and not on a continuous 24-h per day basis. These types of time-to-event data are often referred to as grouped data or interval-censored data [16,18].

This study used the event-time model to analyse the effect of exposure to diesel fuel on the viability and germination rate of *M. sativa* seeds. The event-time model appropriately reflects the experimental design of right-censored germination experiments while allowing the meaningful biological interpretation of germination data.

## 2. Results

### 2.1. Effect of Diesel Fuel on Germination

**Figure 1:**
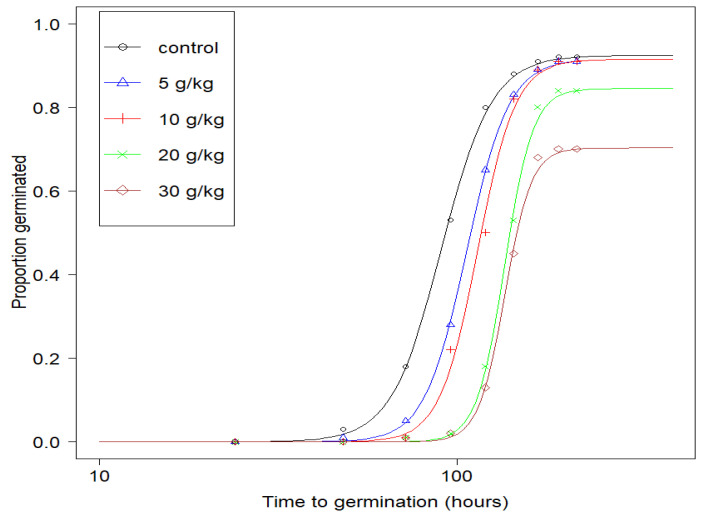
Germination curves for *Medicago sativa* seeds in 0 (control), 5, 10, 20 and 30 g/kg diesel fuel contaminated soils.

The germination curves for *M. sativa* seeds sown in 0 (control), 5, 10, 20 and 30 g/kg diesel fuel contaminated soils are shown in Figure 1. When compared to nonlinear regression models, the event-time model provided the best fit for the germination data. As expected, the proportions of germinated seeds varied with time, being largest at intermediate monitoring intervals (48–120 h for 0 g/kg; 72–144 h for both 5 and 10 g/kg; 96–168 h for both 20 and 30 g/kg diesel fuel-contaminated soils) and smallest at the initial and final intervals when germination activity was low.

Table 1 provides a summary of important germination parameters from Figure 1. These parameters provide insight into the effect that diesel fuel imbibition had on the viability and germination rates of *M. sativa* seeds.

The *t_50_* values for seeds planted in 0 (control), 5 and 10 g/kg diesel fuel contaminated soils were 90.6 ± 2.78, 106.6 ± 2.74 and 114.2 ± 2.67 h, respectively (Table 1). As shown in Table 1, the proportions of seeds in 5 and 10 g/kg diesel fuel-contaminated soils that germinated during the experimental period (indicated by the parameter *d*) were similar to that of the control samples. However, their germination slowed down, as indicated by the higher *t_50_* values. On the other hand, while the *t_50_* values for seeds in the 20 g/kg diesel fuel-contaminated soil remained the same as those for the 30 g/kg soil, the *d* value of the 20 g/kg diesel fuel-contaminated soil was greater than that of the 30 g/kg diesel fuel-contaminated soil. This indicates a possible significant effect of diesel fuel on *M. sativa* seed viability when sown in soils with diesel fuel concentrations of 20 g/kg or more. In addition, the value “1-d” indicates the proportion of the seeds that did not germinate during the experimental period owing to either non-viability or insufficient experimental duration. These seeds were considered to be right-censored.

### 2.2. Effect of Diesel Fuel Exposure on Seed Viability Using Triphenyltetrazolium Chloride

**Figure 2:**
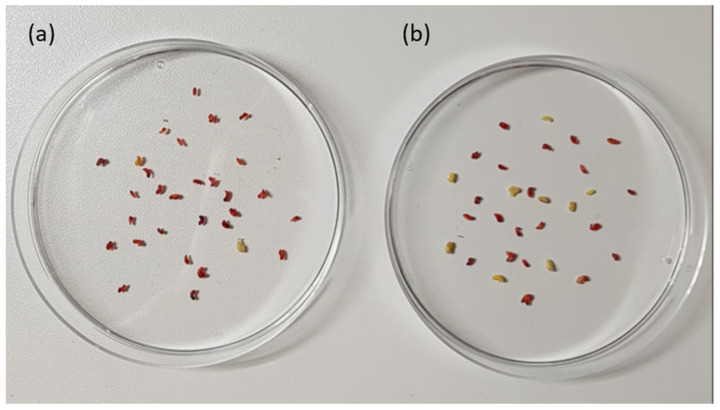
Number of viable *Medicago sativa* seeds, as shown by the red/pink colour of seeds following (**a**) 0 and (**b**) 96 h of diesel fuel imbibition.

**Figure 3:**
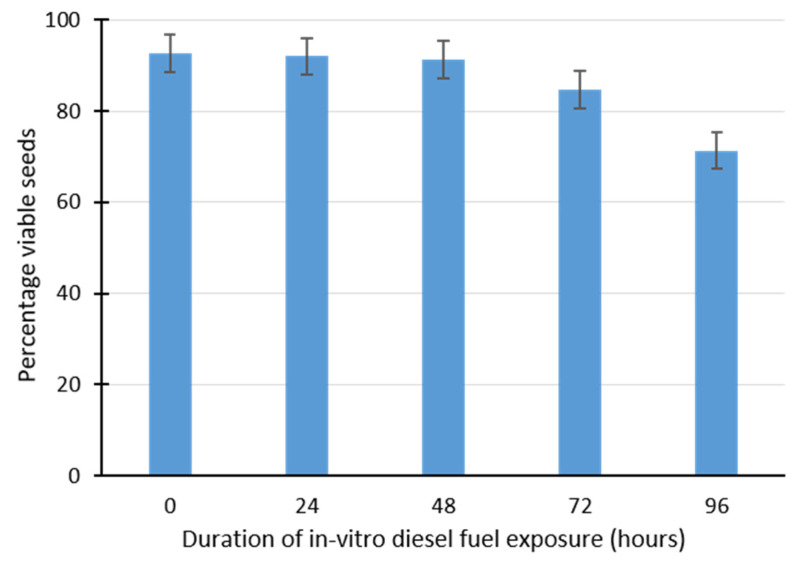
Percentage viability of *Medicago sativa* seeds following in vitro diesel fuel imbibition based on the triphenyltetrazolium chloride test. Error bars indicate standard errors.

The effect of in vitro diesel fuel exposure on *M. sativa* seed viability can be seen in Figure 2. Diesel fuel imbibition reduced seeds viability, as shown by the number of seeds that were stained red or pink during the triphenyltetrazolium chloride test (Figure 2). These results are summarized in Figure 3. The graph indicates that the imbibition of diesel fuel for between 0 and about 48 h had little effect on seed viability. The percentage viability of seeds exposed to 0, 24 and 48 h of diesel fuel imbibition were all greater than 90%. However, exposure of *M. sativa* seeds to 72 and 96 h of diesel fuel imbibition resulted in a decline in percentage seed viability to approximately 84 and 70%, respectively. This indicates that longer duration of direct exposure to diesel fuel affects the viability of *M. sativa* seeds.

## 3. Discussion

This study demonstrates that diesel fuel exposure impacts on the seed viability and germination rate of *M. sativa*, and that these effects are dependent on contaminant concentration and/or duration of exposure. The study also reveals interesting agreement between the grouped-data event-time model and triphenyltetrazolium chloride-dependent viability tests.

To assess the ability of *M. sativa* plants to resist high levels of diesel fuel toxicity, the diesel fuel concentrations used in the germination study were comparable to, or higher than, those used in previous studies involving organic contaminants [19,20,21,22]. Similarly, diesel fuel imbibition was also used to mimic extreme conditions of diesel fuel exposure [23], which enabled us to determine to what extent does extreme exposure to diesel fuel affect *M. sativa* seed viability.

Non-linear regression models are often used to model germination. However, in real life germination experiments, the underlying assumptions governing non-linear models (independence between proportions and variance homogeneity) are not satisfied. In contrast, the event-time model reflects the experimental design of germination experiments and allows meaningful biological interpretation of the germination data. Of course, the restriction F (0) = 0 in the event-time model indicates that the experiment is right-skewed [24]. Thus, it permits log-logistic, log-normal as well as Weibull-type models, which are all models with logarithm transformation [25]. It does, however, rule out models such as the Gompertz, logistic and normal models.

The right-skewness of this model reflects real-life situations in which some seeds do not germinate owing to non-viability or insufficient experimental duration. In these experiments, the seeds viability test using triphenyltetrazolium chloride revealed that *M. sativa* seeds exposed to 24 and 48 h of diesel fuel imbibition have similar percentage viability (92% and 91%, respectively) to that of the control samples. This indicates that the viability of *M. sativa* seeds was unaffected by up to 48 h of diesel fuel absorption. The ability of *M. sativa* to withstand diesel fuel-related biological damage for 48 h is an indication of its potential for biotechnological application in the phytoremediation of diesel fuel contaminated sites. On the other hand, diesel fuel imbibition for 72 h or more impacted seed viability, leading to a decline in the percentage of viable seeds (Figure 3).

The relative resistance of *M. sativa* to diesel fuel toxicity shows its suitability for the rhizoremediation of diesel fuel contaminated sites. It is worth noting that in actual field remediation approaches, seeds are not soaked in diesel fuel. Therefore, we expect that field lethal values (in hours) would be higher than the experimental values from this study. Similarly, actual percentage viability in the field would be expected to be higher than our experimental values. The implication of this is that *M. sativa* is potentially able to survive in diesel fuel contaminated sites. This ability of a plant to withstand contaminant toxicity or similar abiotic stress in the environment is an important factor in designing and establishing successful remediation and reclamation approaches [19].

The results of the germination experiment (Figure 1 and Table 1) provide further biological details. Since the average germination time for *M. sativa* seeds is between two and four days at a temperature range of 18 to 30 °C, the experimental duration was set at nine days to enable the possible germination of all viable seeds. From the results, more than 90% of *M. sativa* seeds exposed to 0 to 10 g/kg diesel fuel contaminated soils germinated during the experimental period, indicating that up to 10 g/kg soil diesel fuel concentration did not affect viability. However, as revealed by the *t_50_* values, soil diesel fuel concentrations impacted significantly on time to germination (Table 1). This indicates increasing time to radicle emergence with increasing concentrations of diesel fuel.

Moreover, seeds exposed to 20 and 30 g/kg of soil diesel fuel concentrations gave lower d values (Figure 1 and Table 1) than those in 0 to 10 g/kg soils. While the proportion of seeds in 20 g/kg and 30 g/kg diesel fuel contaminated soils that germinated during the experimental period varied, their *t_50_* values remained the same (approximately 136 h). This shows that higher concentrations of diesel fuel in soils affects seed viability rather than the time to germination. It can thus be concluded that up to 10 g/kg diesel fuel concentration affects the time to germination of *M. sativa* seeds without significantly affecting their viability. On the other hand, higher concentrations of diesel fuel result in a significant reduction in the viability of these seeds, without further affecting the time required for the seeds to emerge.

The mechanisms by which diesel fuel impacts on seed viability and germination rate can be classified into two: biological damage (toxicity), and physical constraints (oxygen and water repellence). Diesel fuel contains both volatile and non-volatile components [23,26,27]. Previous studies have shown that it is the volatile fraction, rather than the non-volatile components, that is primarily responsible for the inhibition of seed germination and plant growth [23,27], and that at temperatures of <20 °C, this effect is minimal, owing to reduced hydrocarbon volatility [28,29]. *Medicago sativa* seeds in diesel fuel tend to have a lag phase preceding germination (Figure 1), and this lag in germination increases with exposure to increasing diesel fuel concentrations. This can be attributed to the ability of the hydrophobic diesel fuel to create a water-repellent coating around the seeds. This consequently limits both oxygen and water absorption by *M. sativa* seeds, resulting in delayed germination. The fact that diesel fuel at low concentrations and 0–48 h of imbibition was delaying seed emergence without adversely affecting seed viability (as shown by the reduction of triphenyltetrazolium chloride to triphenylformazan) suggests that this inhibitory effect on germination could be attributed more to physical constraints rather than biological damage of the seeds. This is an important quality for biotechnological application since seeds used for phytoremediation purposes must be able to withstand biological damage.

## 4. Materials and Methods 

### 4.1. Soil Preparation

The soil used for this experiment was a mixture of screened sand, soil, and composted organics, sold as “turf underlay” and obtained from Australian Native Landscapes Pty, Sydney, Australia. The soil was sieved using a 2 mm sieve to remove large particles. The soil textural class is dominantly sand (86.2% sand, 5.1% silt and 8.7% clay), with 9.3% organic matter content by loss on ignition and 0.18% total nitrogen content. The soil was then air-dried until a constant weight was achieved. Different concentrations of diesel fuel contaminated soils (0, 5, 10, 20 and 30 g/kg) were prepared by spiking the soil samples with appropriate amounts of diesel fuel. The diesel fuel used was petroleum diesel (also called petrodiesel), as opposed to synthetic diesel or biodiesel, and was obtained from a Shell service station along Epping Road, Macquarie Park, Sydney. The chemical composition is predominantly saturated hydrocarbons (C10 to C25 n-alkanes, iso- and cyclo-alkanes) and some aromatic hydrocarbons (e.g., alkylnaphthalenes and alkylbenzenes). The spiked soils were first mixed manually by hand, followed by a thorough mixing using a Sanfine portable electric 1800 W soil mixing machine (Model No. SF-HM1401/1401S, Taizhou, China). The mixing was performed for 15 min per pot (2 kg soil) with a break and manual shaking after every 5 min to achieve complete homogeneity.

### 4.2. Germination as Grouped Time-to-Event Data

One hundred seeds of *M. sativa* were placed in different 100 mm petri dishes containing 20 g of 0 (control), 5, 10, 20 and 30 g/kg diesel fuel-contaminated soils. The petri dishes were incubated at 20 °C in the Organic Geochemistry laboratory and monitored at 24 h intervals for a period of nine days. The initial emergence of radicle from the seed testa was used as evidence of germination. The seeds that did not germinate during the nine days were considered to be right-censored [17].

### 4.3. Seed Viability Test

In addition to the germination experiment, a triphenyltetrazolium chloride (TTC) test was conducted in petri dishes to estimate seed viability [23,30]. Triphenyltetrazolium chloride is a clear, water soluble compound (a salt) which is reduced by respiring tissues to yield triphenylformazan, a water-insoluble red pigment. Thirty *M. sativa* seeds were pre-soaked in petri dishes containing diesel fuel for 24, 48, 72 and 96 h in order to imbibe seeds prior to the test. At the end of the four respective periods, the imbibed seeds and the control samples were subjected to the triphenyltetrazolium chloride test as follows. A 1% triphenyltetrazolium chloride solution was prepared by dissolving triphenyltetrazolium chloride in distilled water. Each batch of seeds was placed in a beaker containing 50 mL of 1% triphenyltetrazolium chloride, and the beaker was covered. The beakers were placed in an incubator at 30 °C for 1 h. Following incubation, the liquid was decanted and the seeds were rinsed with distilled water until the wash water was clear. The seeds were blotted with a dry paper towel and the colour was observed. The seeds were classified into two categories according to their colour development, namely “red/pink” and “no colour”, corresponding to “viable” and “not viable”, respectively. This test was repeated to give five replicates per treatment concentration.

### 4.4. Statistical Analysis: Event-Time Model

Statistical analyses were performed using R [31]. Non-linear regression models are often used to model germination [32,33]. However, these models are problematic as they ignore the fact that successive observations on the germination curve are highly correlated. In other words, the total number of seeds that have germinated at a particular time is highly dependent on the number of seeds that germinated previously [34]. Moreover, variation in the proportions of germinated seeds will vary with time, being largest at intermediate monitoring intervals and smallest at the initial and final intervals when germination activity is low. This means that the fundamental assumptions underlying nonlinear regression, namely independence between proportions and variance homogeneity, are not satisfied [17]. Consequently, this results in overly precise parameter estimates of, for example, time to reach 50% germination (*t_50_*), due to too small standard errors. Therefore, Ritz and Pipper [17] suggested a more appropriate approach where germination data were modelled as event times, that is, waiting times until germination no longer became possible due to termination of the experiment or non-viability of seeds. This approach provided a more adequate statistical description of the type of response that resulted from germination experiments [35] The present study used the *drm* package in *library(drc)* [31] to model the effect of diesel fuel exposure on seed viability as event-time data. This model is described using the following equation proposed by Ritz and Pipper [17]:

(1) $$F\;\left(t\right)=\;\frac{d}{1+\exp\left[b\left\{\log\left(t\right)-\log\left(t_{50}\right)\right\}\right]}\;=\;\frac{d}{1+\;{\left(\frac{t}{t_{50}}\right)}^{b}}$$

The upper limit parameter *d* denotes the proportion of seeds that germinated during the duration of the experiment out of the total number of seeds present at the beginning of the experiment. The parameter *b* (excluding its sign) is proportional to the slope of *F* at time *t* equal to *t_50_*, while *t_50_* has the same interpretation as effective or lethal doses EC_50_ or LC_50_ but relative to *d* (the upper limit). Thus, *t_50_* refers to the time when 50% of the seeds that germinated during the experiment have germinated. This model reflects the experimental design of right-censored germination experiments while allowing meaningful biological interpretation of germination data. It also links the analysis of germination data with related dose-response analyses used in ecotoxicology where interest lies in obtaining a parametric model fit for an S-shaped curve.

### 4.5. Statistical Analysis: Viability Data

Viability data were analysed as binomial data using the 2-parameter log-logistic model in R [31]. As in the methods of Hose and Symington [36], a series of 2-parameter response curves including log-logistic, Weibull, log-normal and hormetic curves were fitted to the data, and the best fitting model based on Akaike information criterion was chosen. The e value from the model, also referred to as LC_50_, gives the duration of exposure (in hours) causing 50% reductions in viability of the test seed population. The 3-parameter log-logistic event-time model was fitted to the germination data. The event time considered is the time (hours) from sowing to germination, evidenced by the initial emergence of the radicle from the seed testa.

## 5. Conclusions

The examination of the effects of diesel fuel exposure on the viability and germination rate reveals that either the direct absorption of diesel fuel for up to two days or their exposure to soils contaminated with up to 10 g/kg diesel fuel affect germination rate, leading to delayed emergence of *M. sativa* radicle. However, these short durations and low concentrations of exposure to diesel fuel does not affect the viability of *M. sativa* seeds. This is an important quality for biotechnological application since seeds used for phytoremediation purposes must be able to withstand biological damage. Longer duration of diesel fuel imbibition and/or exposure to higher concentrations of soil diesel fuel results in a significant reduction in viable seeds. The event-time model used here provided an accurate and biologically relevant method for analysing germination data. These models incorporate the experimental design of right-skewness and non-continuous observation of germination process. We are confident that the results of this study will prove helpful in the design of plant-based remediation techniques.

## Figures and Tables

**Table 1:** Parameter estimates of the log-logistic model obtained by fitting the event-time model. (Values in parentheses indicate standard errors).

Concentration of Diesel Fuel in Soils (g/kg)	*b* (Slope at *t_50_*)	*d* (Upper Limit)	*t_50_* (h)
0 (control)	−6.16 (0.61)	0.92 (0.03)	90.6 (2.78)
5	−7.41 (0.74)	0.91 (0.03)	106.6 (2.74)
10	−8.26 (0.82)	0.91 (0.03)	114.2 (2.67)
20	−11.12 (1.18)	0.84 (0.04)	135.9 (2.47)
30	−11.87 (1.38)	0.70 (0.05)	136.0 (2.54)
